# Highly absorbent hydrogels comprised from interpenetrated networks of alginate–polyurethane for biomedical applications

**DOI:** 10.1007/s10856-021-06544-4

**Published:** 2021-06-12

**Authors:** Jesús A. Claudio-Rizo, Nallely Escobedo-Estrada, Sara L. Carrillo-Cortes, Denis A. Cabrera-Munguía, Tirso E. Flores-Guía, Juan J. Becerra-Rodriguez

**Affiliations:** 1 Facultad de Ciencias Químicas, Universidad Autónoma de Coahuila, Ing. J. Cárdenas Valdez S/N, República, 25280 Saltillo, Coahuila México; 2 Universidad Politécnica de Pénjamo, Carretera Irapuato - La Piedad Km 44, Pénjamo, 36921 Guanajuato, México

## Abstract

Developing new approaches to improve the swelling, degradation rate, and mechanical properties of alginate hydrogels without compromising their biocompatibility for biomedical applications represents a potential area of research. In this work, the generation of interpenetrated networks (IPN) comprised from alginate–polyurethane in an aqueous medium is proposed to design hydrogels with tailored properties for biomedical applications. Aqueous polyurethane (PU) dispersions can crosslink and interpenetrate alginate chains, forming amide bonds that allow the structure and water absorption capacity of these novel hydrogels to be regulated. In this sense, this work focuses on studying the relation of the PU concentration on the properties of these hydrogels. The results indicate that the crosslinking of the alginate with PU generates IPN hydrogels with a crystalline structure characterized by a homogeneous smooth surface with high capacity to absorb water, tailoring the degradation rate, thermal decomposition, and storage module, not altering the native biocompatibility of alginate, providing character to inhibit the growth of *E. coli* and increasing also its hemocompatibility. The IPN hydrogels that include 20 wt.% of PU exhibit a reticulation index of 46 ± 4%, swelling capacity of 545 ± 13% at 7 days of incubation at physiological pH, resistance to both acidic and neutral hydrolytic degradation, mechanical improvement of 91 ± 1%, and no cytotoxicity for monocytes and fibroblasts growing for up to 72 h of incubation. These results indicate that these novel hydrogels can be used for successful biomedical applications in the design of wound healing dressings.

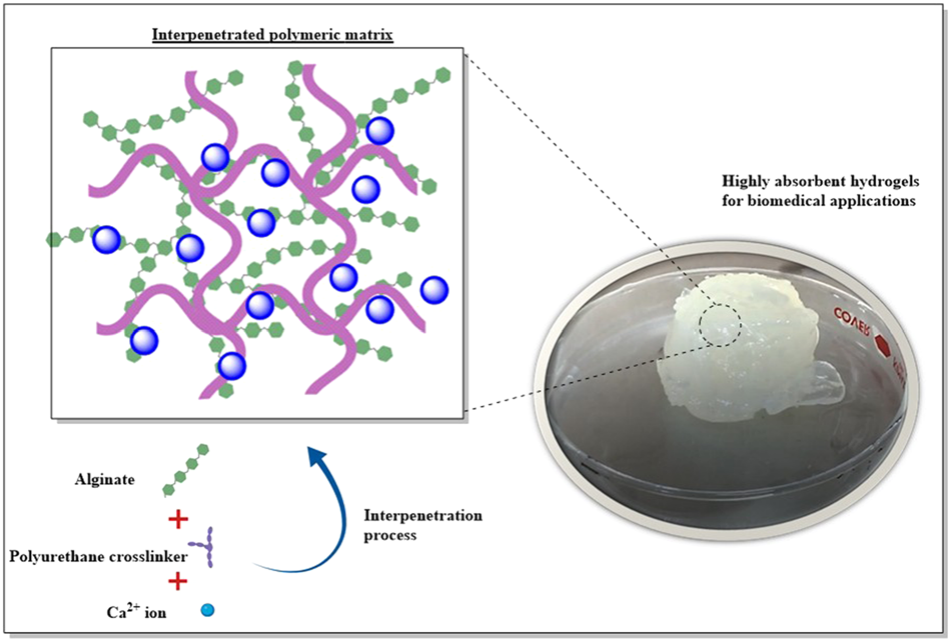

## Introduction

Alginate is a polysaccharide comprised of both guluronic and mannuronic acid units interconnected by β(1 → 4) glycosidic bonds, this is part of the extracellular matrix of different species of algae [1, 2]. Alginate chains have the ability to physically crosslink in the presence of metal ions, such as Ca (II), Mg (II), Ba (II), Zn (II), generating hydrogels with high biocompatibility, they are widely used in the food industry, biomedicine, and tissue engineering [3, 4].

In the biomedical field, alginate hydrogels have been modified by the incorporation of natural polymers (e.g., gelatin [5], chitosan [6], fibrin [7]), synthetic polymers (e.g., polyvinylpyrrolidone (PVP) [8], polyvinyl alcohol (PVA) [9], polyethylene glycol (PEG) [10]), and inorganic agents (e.g., hydroxyapatite [11], zinc (II) oxide [12], silver nanoparticles [13]), tailoring their properties for successful application. Specifically, in the development of wound healing dressings, alginate hydrogels show potential application because they have a high capacity to absorb exudates from the wound, promoting healing [14, 15]; however, there is currently the challenge to develop strategies to improve the swelling capacity, controlling also the degradation rate and mechanical properties of alginate hydrogels enhancing their performance in this sector [16, 17]. The tailoring of these properties should consider strategies that rule out the use of organic solvents and crosslinking agents that tend to decrease the native biocompatibility of alginate; furthermore, the structural modification should allow the generation of hydrogels with desired characteristics to favor the wound healing process, such as the capacity to inhibit bacterial growth and excellent hemocompatibility [18, 19].

In this sense, the generation of hydrogels comprised of interpenetrated networks (IPN) based on alginate with biocompatible synthetic polymers represents an innovative strategy to meet these requirements.

It has been reported that aqueous polyurethane (PU) dispersions allow the generation of IPN hydrogels based on biopolymers like collagen and chitosan with structure, properties, and biocompatibility dependent on the concentration and chemical structure of this crosslinking agent [20, 21]. Furthermore, it has also been reported that these crosslinking agents allow the coupling with inorganic silica particles, providing the controlled release capacity of molecules with interest in biomedical applications [22, 23]. In other words, the use of this crosslinking agent also allows the generation in an aqueous medium of semi-IPN hydrogels employing exogenous polymeric chains such as polyacrylate (PA) and cellulose derived polysaccharides, generating hydrogels with high biocompatibility, enhanced hemocompatibility, and antibacterial inhibition capacity [24, 25].

**Fig. 1:**
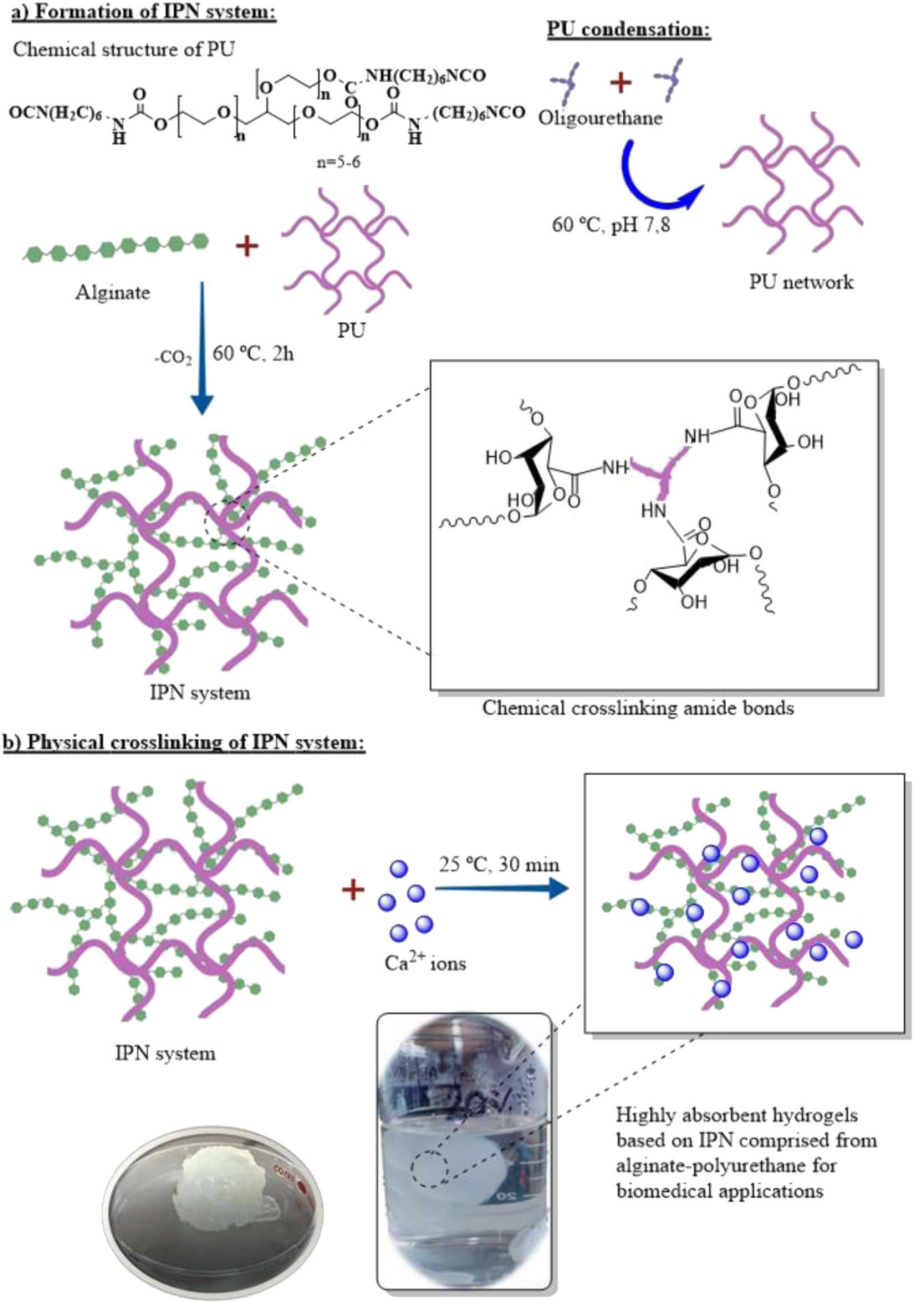
Representative scheme for the generation of novel IPN hydrogels using alginate and aqueous dispersions of PU

Based on this, the present work studies the chemical crosslinking and interpenetration of alginate chains with PU in an aqueous medium to generate novel IPN hydrogels. The isocyanate groups of the PU can be condensed with the carboxylate groups of the alginate to generate interpolymeric crosslinking amide bonds. The generated amide bonds, as well as the polymeric interpenetration, could tailor the swelling behavior, degradation, and mechanical properties of these hydrogels, benefiting their use for biomedical applications. The work focuses on studying the relation of the PU concentration on the structure and properties of these matrices in the hydrogel state (Fig. 1). With this in mind, the work encompasses the synthesis, structural and physicochemical characterization, as well as the evaluation of the in vitro biocompatibility of these novel IPN hydrogels of alginate–PU.

## Experimental section

### Materials

Alginic acid sodium salt (M_n_ 120,000–240,000 g mol^−1^, 61% mannuronic acid and 39% guluronic acid, extracted from brown algae), hexamethylene diisocyanate, glycerol ethoxylate (1000 g mol^−1^), calcium chloride, sodium bisulfite, 3-(4,5-dimethylthiazolyl)-2,5-diphenyltetrazoliumbromide (MTT), phosphate buffer solution (PBS), sodium chloride, sodium hydroxide, Alsever’s solution, Escherichia coli (*E. coli*) bacterial strain, eosin-methylene blue (EMB) agar, and other salts were purchased from Sigma-Aldrich. All reagents were used as received without prior purification processes.

### Synthesis of IPN hydrogels comprised from alginate–PU

#### Preparation of aqueous polyurethane dispersions

The interpenetrating agent of alginate chains was synthesized as previously described elsewhere [21, 23]. Briefly, glycerol ethoxylated was heated to 40 °C and reacted with hexamethylene diisocyanate in a molar NCO:OH ratio of 3:1 for 2 h at 100 °C. The remaining NCO end groups in the polyurethane prepolymer product were reacted with sodium bisulfite (40 wt.% in water) for 2 h at 40 °C, to generate highly soluble carbamoylsulfonate ends.

#### Synthesis of IPN hydrogels

20 mL of alginate solution (1 wt.%) was heated at 60 °C for 30 min; subsequently, different proportions in weight of the PU crosslinking agent were added. Under these reaction conditions, the polyurethane prepolymer condenses to generate a polyurethane-based network that can be chemically interpenetrated with the alginate (Fig. 1a). Table 1 shows the formulations of the hydrogels developed in this work. Alginate is the polymeric component found in the highest proportion in hydrogels to ensure biocompatibility. A maximum concentration of 35 wt.% of PU was established, since higher concentrations of this synthetic agent generate highly insoluble sponges, while concentrations lower than 20 wt.% of PU did not exhibit a significant increment in the viscosity of the system.

**Table 1:** Formulation and designation of synthesized hydrogels

Hydrogel	Alginate content (mg)	Polyurethane content (mg)
Alg	200	0
Alg–PU 20	200	40
Alg–PU 35	200	70

Once the PU was added, the formation of the IPN matrix of alginate–PU in the hydrogel state was carried out by heating at 60 °C for 2 h adjusting the pH to 7.8 using 5 mL of PBS (0.1 M), under constant stirring (400 rpm) (Fig. 1a).

Finally, to induce the physical crosslinking of the alginate chains, 5 mL of CaCl_2_ solution (0.1 M) were added, at room temperature under constant stirring (400 rpm) for 30 min (Fig. 1b). The formed hydrogels were filtered by simple decantation and stored for their structural, physicochemical, and biological characterization. Physical crosslinking was done with calcium ions as a constant condition for all systems. In the case of Alg–PU hydrogels, these are formed without adding the Ca (II) ions; however, the Alg control hydrogel is not formed without this physical crosslinking. With this in mind, it was considered to use Ca (II) as a constant condition in all systems under study. Therefore, the variation in structure and properties of hydrogels is due solely to the concentration of PU.

### Structural and physicochemical characterization

To measure the reticulation index of the hydrogels, acid-base titration was used. For this, 100 mg of each dry hydrogel was taken and mixed in 50 mL of 0.01 M HCl. The system was heated at 60 °C for 30 min and subsequently filtered. The decanted solution was titrated using 0.1 M sodium hydroxide, and phenolphthalein to detect the turning point. The reticulation index was determined based on the ratio of the volumes of titrant (vol. NaOH) for neutralize 100 mg of pure alginate (sample without crosslinking) and the volume used in the hydrogel systems, according to Eq. 1:

1 $$Reticulation\,index\,\left( {\mathrm{\% }} \right) \\ = \frac{{vol.\,NaOH\,used\,in\,hidrogel}}{{vol.\,NaOH\,used\,in\,100\,mg\,de\,alginate}} \ast 100$$

The microstructure of the dry hydrogels (xerogels) was inspected using scanning electron microscopy (SEM), using a Jeol JSM 7600 F microscopy. The samples were covered with a thin layer of metallic gold to ensure the image quality of their microstructure using a Sputter-Coater metallizer. The chemical structure of dried hydrogels was evaluated employing Fourier transform infrared spectroscopy (FTIR), using a diamond attenuated total reflectance (ATR) accessory, for this a Frontier equipment of Perkin Elmer was used, the spectra were recorded with a 16 cm^−1^ resolution in an interval of 4000 to 650 cm^−1^ using an average of 16 scans. The crystalline structure of the interpenetrated alginate–polyurethane systems was also analyzed by Wide Angle X-ray Scattering (WAXS), using a *SAXS-Emc2 Anton Paar* diffractometer with a Cu Kα X-ray source (λ = 1.54 Å). The storage (G‘) and loss (G“) moduli of each type of hydrogel were obtained by oscillatory rheology, using an MCR 300 Anton-Paar rheometer, the samples were analyzed using a 40 mm diameter dish-dish geometry; with a solvent trap to avoid the evaporation of water. All experiments were carried out using 10% of the deformation to ensure the linear viscoelastic behavior of the dynamic response. The thermal behavior of the dried hydrogels was evaluated by thermogravimetric analysis (TGA), a TGA-4000 Perkin Elmer thermoanalyzer was used. The operating conditions used: temperature range from 30 to 800 °C, heating rate of 20 °C/min using nitrogen as an inert atmosphere up to 600 °C (20 ml/min) and oxygen from 600 to 800 °C (20 ml/min).

The mass variation profiles (related to the swelling, degradation, and shrinkage processes of hydrogels) were evaluated by mass variation kinetics in different hydrolytic media (pH 2, 7.4, and 12). For this, a 100 mg sample of each type of dried hydrogel was introduced into 50 ml of each type of study solution. The masses per day of each hydrogel were recorded to observe the swelling and degradation profiles thereof, during a time of 30 days. The mass variation of the hydrogels was evaluated using Eq. 2:

2 $$Mass\,variation\,of\,hydrogel\,\left( {\mathrm{\% }} \right) = \frac{{mass\,at\,time\,t}}{{mass\,at\,t_0}} \ast 100$$

where the mass at time t is the mass of the hydrogel corresponding to the measurement to the day of incubation, and mass at t_0_ is equal to 100 mg.

### Evaluation of in vitro biocompatibility

The variation of metabolism of human monocytes or porcine dermal fibroblasts (got from primary culture) growing on every hydrogel was assessed. Cell suspensions in DMEM culture medium were added to the wells containing hydrogels (50,000 cells/well) and control (without hydrogels), and these were incubated for 12, 24, and 72 h. The cells were inoculated on the surfaces of the hydrogels (200 mg) and allowed to incubate for the times mentioned. Independent experiments were used for each type of cell. The cell viability was determined by the ratio of cells with dynamic metabolism to transform MTT salts in formazan growing on hydrogels, with respect to control. The MTT (1% w/v) was added to wells with hydrogels and controls, and afterward, the cells were kept up under culture conditions for 3 h at 37 °C. At that point, the medium was tapped, the blue formazan was diluted in isopropanol, and the absorbance of the solutions was estimated at 560 nm, using a Synergy HTX Multi-Mode Reader Biotek spectrophotometer. The absorbance of generated formazan by cells growing in wells without hydrogels is equal to 100% of viability (controls).

The hemolysis test was performed to determine the hemocompatibility of the hydrogels. This test measures the hemoglobin released when the red blood cell membrane is destroyed. A human blood sample was obtained, then it was centrifuged at 3000 rpm for 5 min, the plasma was decanted and the erythrocytes were obtained. Red blood cells were washed with 5 mL of Alsever’s solution. Then, 100 μL of purified erythrocytes were taken and diluted in 10 mL of Alsever´s solution. A quantity of 150 µL of this solution was added in tubes containing 5 mg of each dry hydrogel, in addition 1850 µL of Alsever’s solution was added too. The tubes were incubated with shaking at 37 °C for 30 min. Finally, the tubes were centrifuged to measure the released hemoglobin at a wavelength of 415 nm. The hemolytic capacity (%) of hydrogels was calculated by the ratio of the absorbance value obtained for hydrogels with respect to absorbance values of positive control (water) and negative control (Alsever´s solution).

In vitro bacterial inhibition tests were performed using EMB agar and a bacterial strain of *E. Coli*. The agar was prepared by dissolving 37.5 g of dehydrated agar in 1 L of distilled water and was heated until dissolved. The culture medium was sterilized between 121–124 °C for 15 min. Once the medium was ready, it was poured into Petri dishes which were previously sterilized. Dry hydrogel samples were placed in the center of the culture medium dishes, with the finality to measure the inhibition halo. Doxycycline prepared at 20 ppm was used as a control. The bacterial strain was seeded homogeneously in the Petri dishes, including hydrogels and control using a sterile loop. The bacterium was incubated for 24 h at 37 °C to measure its growth in the presence of the hydrogels. The inhibition capacity of *E. Coli* was calculated by comparing the diameter of the halo formed by the hydrogel with respect to the diameter generated by the control.

### Statistical analysis

The experiments were done in triplicate. The values of mean and standard deviation are considered for each experiment. Data means were compared using one-way analysis of variance (ANOVA). The difference of the means was checked with a Tukey test and was considered statistically significant at level *p* < 0.05.

## Results and discussions

### Structural and physicochemical characterization

**Fig. 2:**
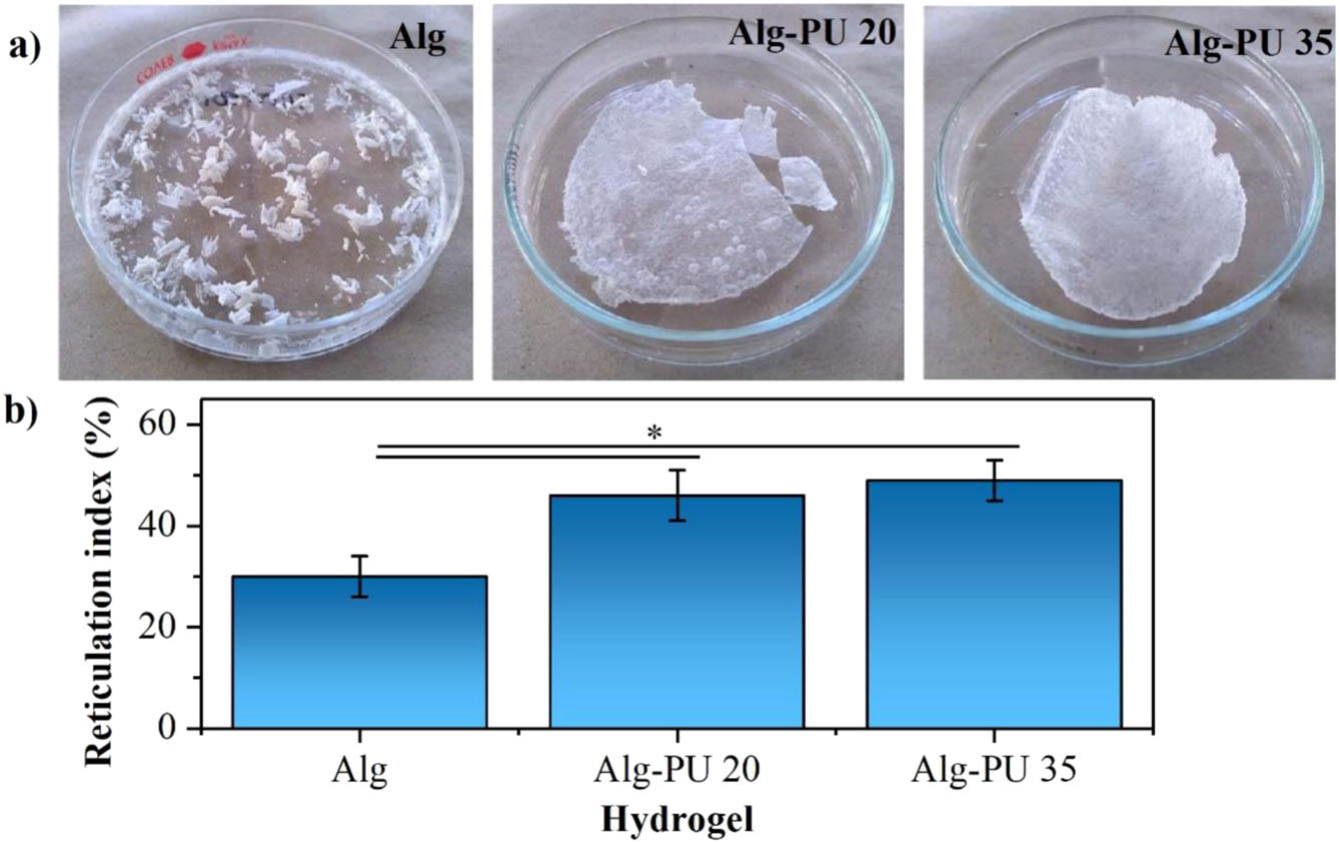
**a** Appearance of studied xerogels and (**b**) determination of the reticulation index by acid-base titration

PU dispersions can generate IPN hydrogels with alginate chains in an aqueous medium when this synthetic polymer is used in concentrations ranging from 20–35 wt.%. In this concentration range of PU, a significant increment in viscosity is observed, indicating the formation of an IPN matrix in the hydrogel state; the appearance of the dried hydrogels (xerogels) obtained is shown in Fig. 2a.

The alginate hydrogel (Alg) shows a brittle texture with very poor manageability; in the case of hydrogels interpenetrated with PU (Alg–PU 20 and Alg–PU 35), homogeneous xerogels with high manageability are obtained, their capacities to withstand deformation is higher than Alg hydrogel, since the alginate chains are crosslinked and interpenetrated with the PU chains significantly improving their manageability.

The increment in the PU concentration increases the rigidity of the obtained xerogels. By increasing the concentration of PU the material becomes more opaque; however, Alg–PU 20 hydrogels have good transparency that can be used for applications such as dressings. By using concentrations higher than 35 wt.% of PU, segmented PUs based on alginate are obtained characterized by having a sponge structure, this type of material has other characteristics and properties for biomedical applications different from hydrogels based on alginate [26, 27].

In Fig. 2b, the values of the reticulation indices determined by the quantification of the free carboxylic acid groups of the alginate chains with and without interpenetration with PU are presented. The values determined are 30 ± 4, 46 ± 5, and 49 ± 4%, for Alg, Alg–PU 20, and Alg–PU 35, respectively. The carboxylic acid groups involve important crosslinking interactions with calcium (II) ions and intermolecular, generating the hydrogel state [1–4]. Statistically, significant differences are found when comparing the reticulation value of the Alg hydrogel with the IPN hydrogels. The carboxylate groups of the alginate form chemical crosslinking bonds of amide nature with the isocyanate-reactive ends present in the aqueous dispersions of PU, during the condensation process, thus generating an IPN matrix. These amide bonds are stable products of the decarboxylation of the generated anhydrides by the addition of the alginate carboxylate with the isocyanate group of the PU. Different crosslinkers to form alginate hydrogels with adapted properties have been used, such as glutaraldehyde [28, 29], and carbodiimides [30]. In the case of PU, it has also shown a direct relation in the modulation of the crosslinking index with its concentration in hydrogel systems for biomedical applications based on collagen [20, 21]. In this way, this proposed approach represents a favorable method in an aqueous medium for the control of the reticulation of reactive alginate to isocyanate groups to generate IPN hydrogels.

Based on the concentrations of used PU, no significant differences could be determined in the crosslinking indexes of the IPN matrices; this may be associated with aspects of matrix formation in the hydrogel state. High crosslinking indices (higher than 60%) generate materials such as segmented alginate-based PU which are characterized by having a sponge structure. Concentrations higher than 35 wt.% of PU produce higher crosslinking indices with alginate, generating insoluble sponges that lose the ability to absorb water, not representing systems in the hydrogel state.

**Fig. 3:**
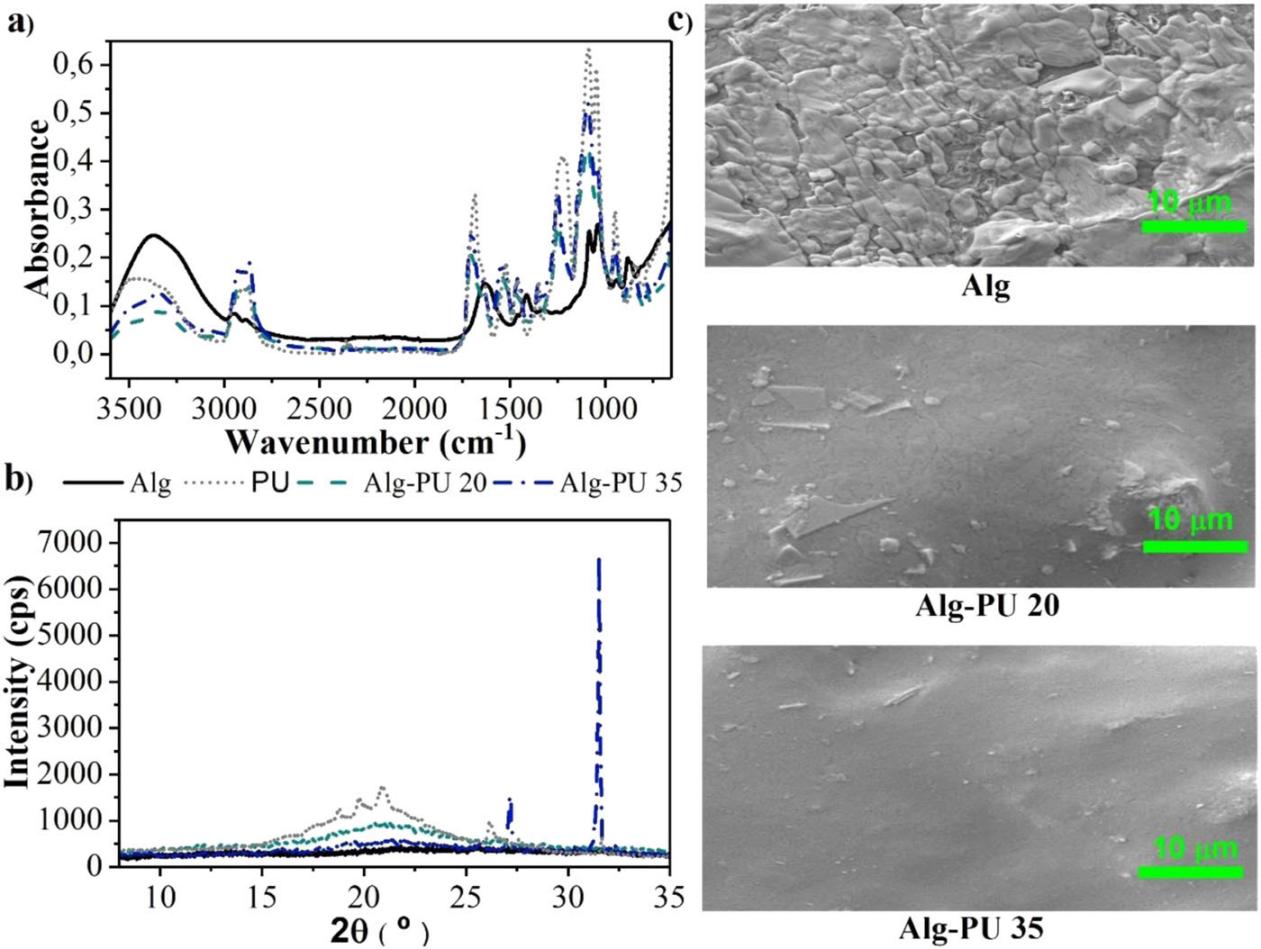
Evaluation of the (**a**) chemical structure by FTIR, (**b**) crystal structure by WAXS and (**c**) microstructure by SEM of the IPN hydrogel-based alginate–PU

The modification of the chemical structure of alginate by the crosslinking and interpenetration of the PU chains was evaluated by FTIR; the spectra of the hydrogels are shown in Fig. 3a. The Alg hydrogel shows stretching bands at 3372, 2949, 1636, 1410, and 1085 cm^−1^ related to the hydroxyl bonds (–OH) of the alcohol, –CH of the glucosidic skeletons, C=O of the alginate carboxylate, C–O, and C–O–C characteristic of polysaccharides, respectively [28]. In the FTIR spectra of the Alg-PU hydrogels, the alginate carboxylate band at 1636 cm^−1^ is no longer seen, indicating that this functional group is directly related to the formation of new amide bonds. Crosslinking amide bonds are observed at 1610 cm^−1^ in IPN hydrogels. The pKa values for the carboxylic acid and alginate–OH are 4 and 13, respectively. These values indicate that at the pH at which the hydrogels were designed (pH 7.8), the carboxylate groups are deprotonated, being better nucleophiles to condense with the PU isocyanates generating amide bonds, compared to the –OH groups of the alginate skeleton that have poor nucleophilic character. In the condensation reaction among an isocyanate group and a carboxylate, an intermediate carbonic anhydride is produced. This anhydride decomposes releasing CO_2_, generating crosslinking amides bonds among the PU and the alginate. The amide bands observed by FTIR in the IPN systems confirm this mechanism of IPN hydrogel formation.

The decrease in the signal intensity of the –OH bonds at 3372 cm^−1^ in IPN hydrogels indicates that the polymeric interpenetration process originates short-range interactions of the hydrogen bridge type, directly evidencing the formation of IPN systems. The urethane bond band for PU is around 1680 cm^−1^ with higher intensity. In the Alg–PU IPN hydrogels indicate a displacement of the urethane bond band at 1697 cm^−1^ with a decrease in intensity, evidencing the formation of an IPN system.

The signals related to the glycosidic backbones in the spectra of the hybrid hydrogels indicate that the PU chains are able to chemically and physically crosslink the alginate chains, forming a hydrogel system of IPN networks. The modification of the intensities of the signals for the C–O–C bonds between 1250–1000 cm^−1^ indicate direct evidence with the crosslinking and/or physicochemical reticulation among the two polymeric agents; that is, system with highest crosslinking and interpenetration exhibits the highest intensity of these signals, as seen for the hydrogel with 35 wt.% of PU. The detection of reticulation bonds and their dependence on the concentration of the reticulation agent is decisive for developing IPN hydrogels with adapted properties. Previously, it has been reported that this synthetic agent shows direct evidence of urea crosslinking bond formation when generating hydrogels comprised from collagen–PU [20, 21].

The presence of crystalline phases in the prepared hydrogels was evaluated by WAXS, in Fig. 2b the diffractograms of the materials are shown. The alginate-based hydrogel has an amorphous structure without diffraction signals that prove crystalline planes within its chemical structure. PU shows diffraction signals at 18°, 20°, 21°, and 26° associated with a structure with low crystallinity. The Alg–PU 20 and Alg–PU 35 IPN hydrogels present signals with significant intensity at 2θ equal to 21°, 27°, and 32°; evidencing that the chemical crosslinking and interpenetration of the alginate chains with the PU generate regions with structures with crystalline order. By increasing the concentration of PU, the signals at 27° and 32° increase in intensity, indicating that the chemical crosslinking by amide bonds increments the crystallinity of the Alg-PU IPN hydrogels, as seen in the Alg–PU 35 hydrogel.

It has been shown that the generation of interpenetrating polymeric matrix systems generates regions with characteristic crystallinity, this is associated with the crosslinking, entanglement, and interpenetration interactions of polymeric agents producing regions with molecular order that can diffract X-rays [24, 25, 31]. Hydrogel systems based on IPN networks with crystalline regions provide sites with selective modulation in cellular processes optimizing the biological response in biomedical applications; cell–material interaction is important to regulate cell metabolism; in this way, cells that adhere to crystalline or amorphous surfaces would experience characteristic stimuli that affect their metabolism and secretion of important cytokines in the healing process [32]; this could be beneficial to favor the wound healing processes if these matrices are applied as wound healing dressings.

The microstructure of these novel hydrogels was studied by SEM, the micrographs of these systems are provided in Fig. 3c. Alg hydrogel shows a surface microstructure with a granular appearance, the texture is rough and these granules do not have a homogeneous size, which is characteristic for alginate [33]. In Alg--PU 20 hydrogel, its microstructure exhibits a surface with a moderately smooth and homogeneous texture, the characteristic granules of Alg disappear almost entirely; finally, the microstructure for the Alg–PU 35 hydrogel presents a completely homogeneous and smooth surface. The interpenetration of the linear alginate chains with the PU elastomeric agent generates structures with surface homogeneity; thus evidencing that polymeric interpenetrating amide bonds provide crystalline order to alginate hydrogels as confirmed by WAXS. This type of surface can be used to make bandages in the hydrogel state that are attached to the wound bed, ensuring biomedical efficacy due to the exhibited surface homogeneity [13–16].

**Fig. 4:**
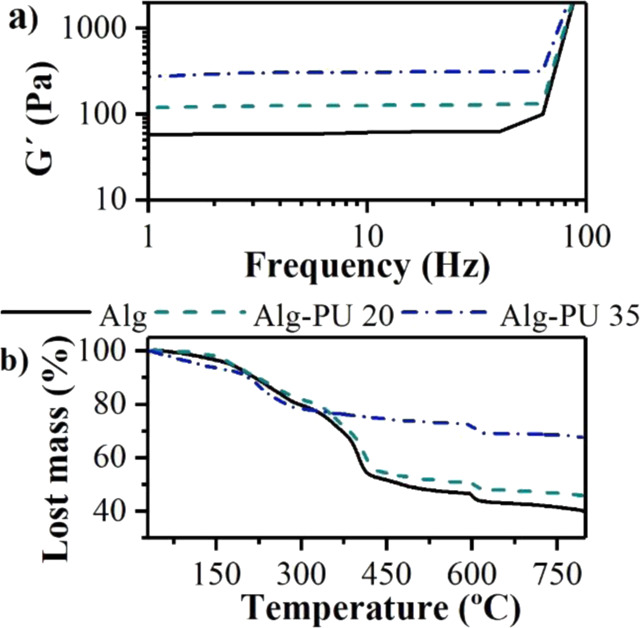
Determination of (**a**) Storage module by oscillatory rheology and (**b**) thermal degradation evaluated by TGA for hydrogels under study

The variation of the mechanical behavior of the alginate hydrogels interpenetrated with PU was evaluated by oscillatory rheology. The studied hydrogels presented storage modulus values higher than the loss modulus, indicating that these matrices are characterized by having a viscoelastic behavior associated with the hydrogel state. The storage modules of the hydrogels under study are shown in Fig. 4a.

The obtained G’ at an oscillation frequency of 10 Hz for Alg, Alg–PU 20, and Alg–PU 35 is 54 ± 2 Pa, 103 ± 3 Pa, and 280 ± 2 Pa, respectively. Indicating that a mechanical improvement of 91 ± 1 and 418 ± 3% are registered for Alg–PU 20 and Alg–PU 35, respectively, this comparing with the G´ value of the hydrogel without interpenetration with PU. The crosslinking and interpenetration of the alginate chains with the PU chains obtains a notable change in the capacity in which the matrix supports mechanical loads, significantly improving the storage modulus, this relation is also dependent on the PU concentration.

The presence of the reticulation amide bonds among the carboxylate groups of the alginate with the isocyanates of the PU is responsible for this significant mechanical improvement, as well as the physical crosslinking and entanglement that exists in this IPN system, regulated by hydrogen bonds among the –OH groups of the polysaccharide backbones and the –NH of urethane bonds abundant in the matrix. The WAXS results also confirm that the material with the highest crystallinity is the one that significantly improves its storage modulus (Alg–PU 35), indicating that these short-range interactions are involved in increasing the storage modulus of these IPN matrices in the hydrogel state. From the point of view of application as wound healing dressings, this mechanical improvement can be used to avoid the contraction of the hydrogel induced by the growth of the cells involved in the healing process, optimizing its period of use for a successful application [34]. Various works have reported various strategies to improve the mechanical properties of alginate hydrogels to enhance their biomedical performance [10–12, 30–32]. Also, the G´ improvement of alginate hydrogels can be used for the encapsulation of molecules with therapeutic interest such as anti-inflammatories, vasodilators, vasoconstructors, dermoregenerators; without compromising the structure of the hydrogel, thus improving its performance in wound healing. The encapsulation of therapeutic agents within matrices in the hydrogel state requires improved storage moduli to ensure the mechanical integrity of the 3D matrix avoiding the deformation, also allowing a controlled release thereof [10–12, 22].

The thermal degradation behavior of the hydrogels was analyzed by TGA; in Fig. 4b the thermograms obtained for this work are shown. Thermograms are characterized by having 3 stages of mass loss: from 25 to 120 °C the dehydration of the hydrogels is presented, from 200 to 550 °C the endothermic decomposition of organic matter is observed, generating gaseous by-products that decrease the mass of the hydrogels, and finally, from 600 to 800 °C the exothermic decomposition is fostered for the formation of inorganic ashes under the action of oxygen. Taking as a reference the endothermic decomposition of organic matter at 400 °C, there are mass losses of 51 ± 3% in Alg hydrogel, 45 ± 4% in Alg–PU 20 hydrogel and 25 ± 3% in Alg–PU 35 hydrogel. According to these data, an improvement in resistance to thermal degradation of 12 ± 3 and 51 ± 4% are calculated for Alg–PU 20 and Alg–PU 35, respectively, compared with the Alg hydrogel. To increment the PU concentration also improves the resistance to thermal degradation. The increment in the reticulation index, as well as the generation of the IPN alginate–PU matrix, generates regions with crystal order characterized by resisting the thermal degradation requiring more heat to remove the short-range interactions such as intermolecular hydrogen bonds (associated with molecular entanglements) and crosslinking bonds, this is strong evidence for the characterization of an IPN system [35]. This improvement in resistance to thermal degradation is in accordance with the increment in the crystallinity and mechanical improvement, as has been reported in various works where other biopolymers are modified [24, 25].

**Fig. 5:**
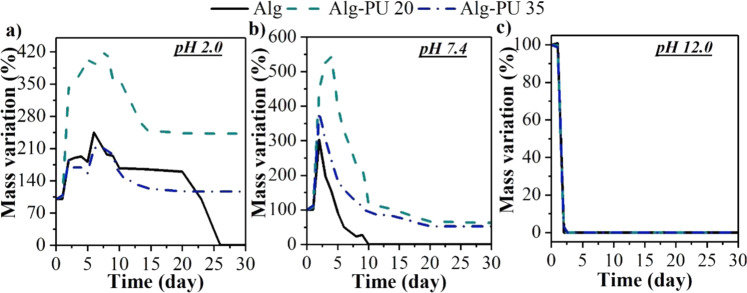
Evaluation of mass variation profiles of hydrogels by varying the pH: (**a**) acid pH, (**b**) physiological pH, and (**c**) alkaline pH

Finally, in the physicochemical characterization, the mass variation profiles of the hydrogels on different hydrolytic media (pH 2.0, 7.4, and 12) were evaluated; in order to study their swelling, degradation and/or shrinkage behaviors. The results are seen in Fig. 5.

Waste metabolites are produced in the wound bed, these tend to acidify the pH of the medium, this change in pH is responsible for altering the structure of the hydrogel causing its disruption; therefore, studying the effect of pH on the swelling, degradation, and/or shrinkages behaviors of these IPN hydrogels represents a key factor for a successful performance [36].

At acidic pH (Fig. 5a), maximum swelling capacities of 245 ± 11% at 7 days, 421 ± 16% at 10 days, and 212 ± 9% at 8 days, are exhibited for the Alg, Alg–PU 20, and Alg–PU 35 hydrogels, respectively; later, the IPN matrices with PU undergo diffusion of the absorbed water, decreasing the swelling at 236 ± 14 and 106 ± 16% for Alg-PU 20 and Alg–PU 35 after 30 days of incubation at this pH; while that the alginate matrix shows complete mass loss at 26 days of incubation; which is caused by the acid hydrolysis reactions that produce the decomposition of this matrix, benefiting the degradation and shrinkage of the hydrogel. In this sense, the interpenetration and crosslinking of alginate with PU produce hydrogels with resistance to lose mass in acidic conditions. The amide bonds that keep the interpenetrated polymeric chains delay the mass loss at the time evaluated. Furthermore, statistically significant differences are determined when comparing the swelling values of the Alg–PU 20 hydrogel with respect to the Alg and Alg–PU 35 hydrogels; indicating that at this PU concentration the swelling capacity is enhanced; generating a surface with permeability and capacity to absorb high masses of water.

At physiological pH (Fig. 5b), the IPN matrices exhibit higher swelling capacities than at acid pH, registering values of 302 ± 16% at 3 days, 368 ± 19% at 4 days and 549 ± 18% at 7 days, for Alg, Alg–PU 20 and Alg–PU 35, respectively. Later, the alginate hydrogel undergoes total mass loss at 10 days of incubation; while interpenetrated systems with PU report mass losses of 25 ± 4 and 38 ± 6% for Alg–PU 20 and Alg–PU 35%, respectively, after 30 days of incubation. These improvements in the swelling/mass loss behavior depend on the bulk material, generating high water-absorbent matrices. The presence of the crosslinking amide bonds, as well as the rich composition in ethoxy groups of the PU are responsible for this improvement in the swelling capacity, however, there is no direct relation between the concentration of PU with the increment in the capacity of swelling; this is associated with the fact that high concentrations of PU generate matrices with higher crystallinity that tend to decrease the permeability and diffusion of water [20, 25]. By the other hand, this interpenetration with PU delays that the alginate being hydrolytically degraded and/or shrunken at neutral pH, thus improving the application time of these hydrogels for potential use as wound dressing dressings, allowing also the removal of wound exudates.

By increasing the concentration of hydroxyl ions, at alkaline pH, it can be seen that the matrices completely lose their mass after 2 days of incubation under these conditions (Fig. 5c), indicating that IPN hydrogels are highly susceptible to hydrolytic degradation/shrinkage in basic medium. Hydroxyl ions tend to hydrolyze the amide crosslinking bonds, as well as the glycosidic bonds of the alginate, disintegrating the matrices in the hydrogel state. These results suggest that these matrices would not have useful applicability in systems with alkaline exudates [36]; since the disintegration of the matrix would begin before the wound healing [37]. On the other hand, knowing the mass variation profiles of these novel matrices allows to visualize their potential application for the encapsulation and controlled release of therapeutic agents by modifying the pH. In this sense, several drug encapsulation studies with biomedical interest and their release profiles have been reported using alginate hydrogels [38]. With this in mind, these novel matrices that exhibit diffusion-induced swelling of water molecules into the hydrogel, could release an encapsulated drug in a controlled manner as water enters inside the matrix; representing devices in the hydrogel state with the capacity for controlled release.

### Studies of in vitro biocompatibility

**Fig. 6:**
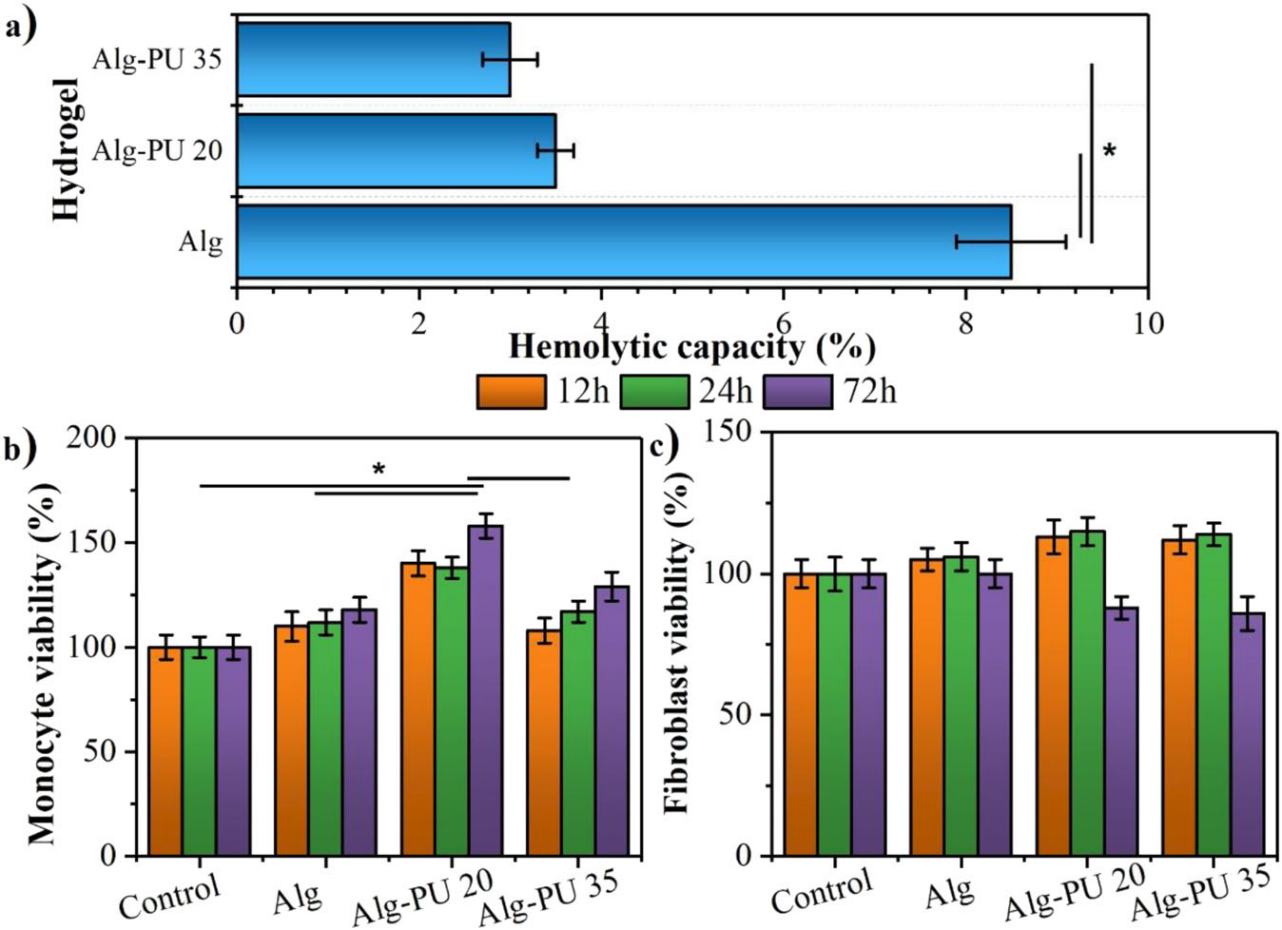
In vitro biocompatibility: (**a**) Hemolysis tests using erythrocytes and metabolic activity evaluated by the MTT test for (**b**) monocytes, and (**c**) fibroblasts

The modification of the structure and physicochemical properties that the alginate undergoes to generate these IPN hydrogels with PU directly could influence its biocompatibility; therefore, studying the effects that this interpenetration process produces on the behavior of cells with interest in the wound healing process represents a fundamental objective so that hydrogels can be applied in biomedical strategies. The in vitro experiments using cells on IPN hydrogels have the objective of allowing to evaluate the modification of the biocompatibility of alginate. In Fig. 6, the results for the in vitro biocompatibility tests carried out are shown.

To determine the hemocompatibility of the materials with blood, the hemolysis test was performed using human erythrocytes. If the composition of the degradation products and the structure of the hydrogels that come into contact with these cells tend to modify their membrane and break it down, then these cells die and release the hemoglobin that they carry. The results for this test are shown in Fig. 6a. Hemolytic capacities of 8.2 ± 0.7, 3.7 ± 0.3, and 2.5 ± 0.3% are determined for the Alg, Alg–PU 20, and Alg–PU 35 hydrogels, respectively. Statistically significant differences are found when comparing IPN hydrogels with PU with respect to alginate hydrogel alone; indicating that the presence of this synthetic interpenetration agent decrements the hemolytic capacity of the alginate, improving its hemocompatibility. According to the ASTM F 756-08, IPN materials are classified as non-hemolytic hydrogels, thus they could be easily applied to the wound beds, avoiding blood lysis. It has been reported that the interpenetration of biopolymers with this type of crosslinking agent increases the hemocompatibility of hydrogels developed for biomedical applications [24, 25]. The flexible structure of PU and the ability it provides to the IPN matrix to absorb high amounts of water prevents the erythrocyte cell membranes from breaking, improving hemocompatibility. Hydrogels with high swelling capacity have been reported to prevent cell lysis due to diffusion control of water molecules from the hydrogel-liquid interface. On the other hand, the control of the disintegration that the PU has on the matrix, benefits that fewer degradation products are releasing not altering the ionic strength of the medium significantly, avoiding the lysis of erythrocytes; in this sense, the degradation products can interact with the cell membrane and alter its ionic and osmotic balance, favoring its breakdown.

Studying the effect of the degradation products of hydrogels on cell metabolism is important since it allows monitoring if there are cytotoxic events that prevent that important cells in the wound healing process from growing and proliferating on these matrices; or if the composition alters their metabolism, which may promote metabolic routes that influence tissue remodeling [39, 40]. The evaluation of the modification of the cellular metabolism of monocytes growing on these hydrogels was determined by the MTT assay. The results are displayed in Fig. 6b. Monocytes are important cells of the immune system that are responsible for the internalization of macrophages and fibroblasts that regulate the processes of inflammation and tissue reconstruction, respectively [40, 41]. Based on the results, all the hydrogels exhibit monocyte viability values higher than 80% at the evaluated culture times, indicating that these matrices do not present a cytotoxic character for monocytes. Statistically significant differences are determined by comparing the monocyte viability values at 72 h of Alg–PU 20 with respect to the other hydrogels and the control; indicating that this composition tends to increase the metabolic activity of monocytes to reduce the salts of MTT to formazan. The structure of this novel hydrogel, as well as its improved swelling capacity, influences the metabolic activation of monocytes; this can be taken advantage of to generate wound healing strategies with shorter healing times. It has been reported that various chronic wounds require that monocytes be metabolically activated to benefit the healing process [16, 40]; in this sense, the modulation of monocyte metabolism via the composition and structure of the hydrogel represents an innovative strategy for a successful biomedical application.

The effect of hydrogel composition on fibroblast metabolism is presented in Fig. 6c. For the three evaluated incubation times, fibroblast viabilities higher than 80% were determined, also indicating that the structure and composition of the hydrogels studied do not represent a cytotoxic character for these important cells in tissue remodeling. Fibroblasts can breathe actively in the presence of degradation products derived from these hydrogels, without significantly influencing the composition of the hydrogel with respect to fibroblasts growing without the presence of hydrogels (controls); reducing similar MTT concentrations to formazan for all compositions at incubation times of 12 h and 24 h; however, at 72 h there is a decrease in fibroblast viability in IPN hydrogels; this could be associated to the fact that the degradation products of these hydrogels modulate the metabolic activity of fibroblasts at long incubation periods, and this modulation could influence that this type of cells tend to generate cytokines associated with the construction of new tissue, such as has been reported for various biomedical strategies involving fibroblasts for wound healing [41, 42].

**Fig. 7:**
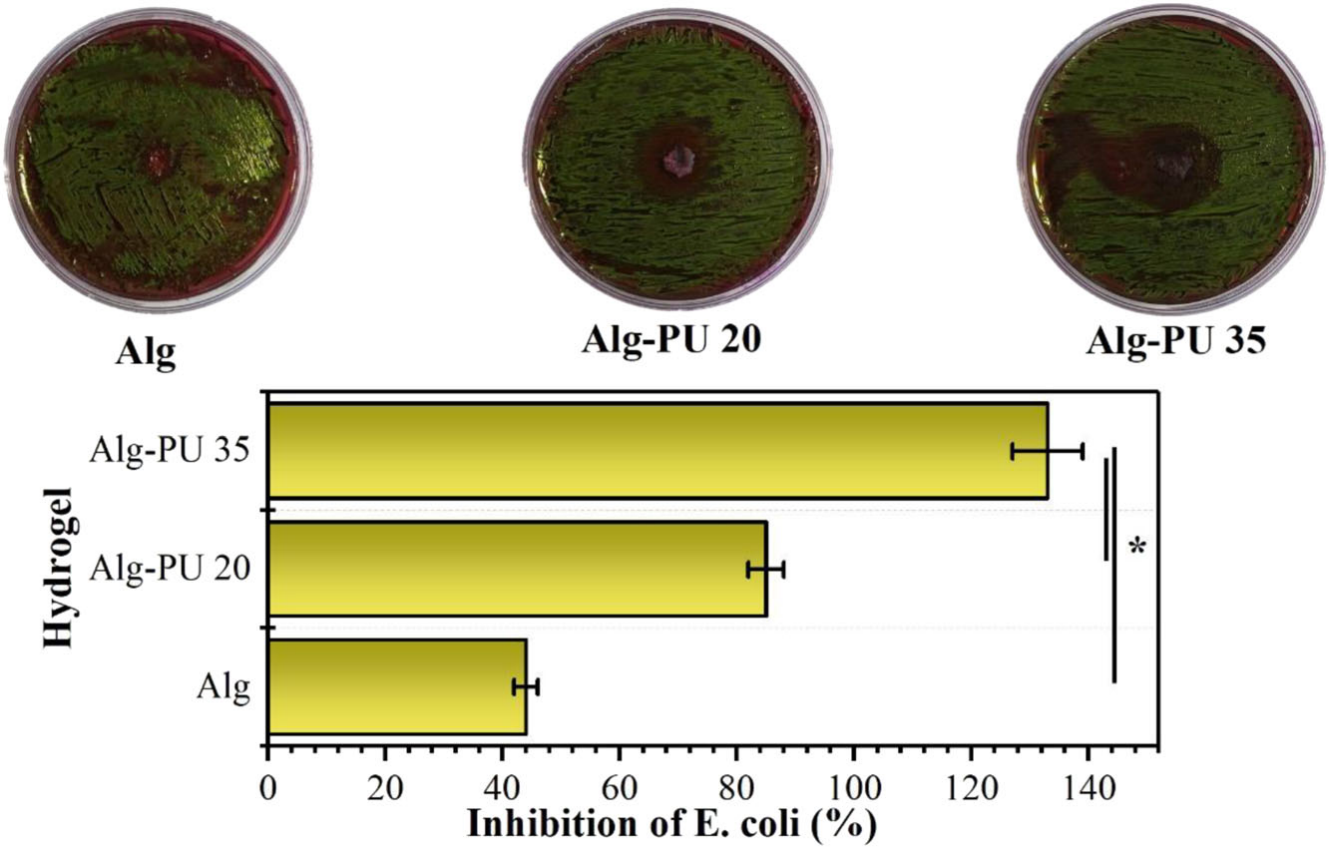
Results of the test of *E. coli* inhibition halo in the presence of the alginate-PU IPN hydrogels

On the other hand, an important characteristic that a hydrogel should possess for potential application as a wound healing dressing is that it has the ability to inhibit the growth of bacteria that develop infections in the wound bed. Chronic wounds make healing difficult due to the presence of these infections [43]. The *E. coli* bacterium is a microorganism that tends to symbiosis with other types of bacteria associated with infections in soft tissue wounds such as *S. aureus* [43]; therefore, developing strategies that prevent this microorganism from growing on the matrices in the hydrogel state would enhance its biomedical application. To evaluate whether the developed hydrogels have this property, the halo of inhibition test was used with an *E. coli* strain. The results are shown in Fig. 7.

Colonies of *E. coli* bacteria grow in the presence of EMB medium, forming a characteristic metallic green coloration. The obtained antibiograms show the formation of inhibition halos (regions where *E. coli* colonies did not grow) in the culture medium in the presence of the studied hydrogels; these halos grow in diameter when increasing the concentration of PU reticulation agent. The inhibition capacities of *E. coli* at 24 h of incubation, determined with respect to the reference control (doxycycline), are 43 ± 4, 84 ± 6, and 132 ± 9% for Alg, Alg–PU 20 and Alg–PU 35, respectively. Statistically, significant differences are found when comparing the inhibition value of the Alg–PU 35 hydrogel with respect to Alg and Alg–PU 20 hydrogels. Thus, a proportional relation between the capacity to inhibit the growth of *E. coli* and the concentration of PU is determined; indicating that these IPN hydrogels exhibit enhanced inhibition capacity of this bacterium.

The surface modification that the alginate experiments with the reticulation and interpenetration produced by the PU is responsible for preventing the growth of bacteria in the agar, showing a clear anti-bacterial effect, and that the leachates from the hydrogel also impede their growth. It has been reported that the chemical structure of alginate prevents bacterial growth, therefore it is widely used in the food industry and in biomedical controlled release devices; this is mainly associated with the presence of Ca (II) ions and polysaccharide skeletons with a specific structural configuration that inhibits bacterial growth [44]. Other cellulose-derived polysaccharides such as starch have also provided the ability to inhibit bacterial growth in hydrogels for biomedical applications [24]. Furthermore, the use of this synthetic crosslinking agent in hydrogels based on semi-IPN collagen with polyacrylate has shown to provide the capacity to inhibit the growth of *E. coli* [25]. Increased crystallinity of IPN hydrogels is also an indication of increasing bacterial inhibition character [45].

Since hydrogels show the capacity to inhibit the growth of pathogenic bacteria, it would not be necessary to use exogenous antibiotics in the wound bed. Therefore, hydrogels based on interpenetrated alginate–PU networks presented in this work that show potential bacterial inhibition, could be very useful for successful biomedical application, specifically as wound healing dressings.

## Conclusion

The interpenetration and reticulation of alginate chains with PU, in an aqueous medium, generates hydrogels with structure and physicochemical properties tailored with the concentration of this synthetic polymer. Concentrations up to 35 wt.% allow to obtain materials in the hydrogel state, without modifying the native biocompatibility of the alginate. The control of the structure and properties is influenced by the generation of amide bonds that influence the reticulation and interpenetration capacity of the polymeric components to generate these novel matrices in the hydrogel state. The hydrogel containing 20 wt.% of PU exhibits a high capacity to absorb water, improved storage modulus, slower degradation rate in neutral and acidic media; also showing improved hemocompatibility and bacterial inhibition capacity, not representing cytotoxic character for monocytes and fibroblasts up to 48 h of contact. Due to this, these hydrogels can be excellent materials for potential application as dressings for wound healing since they cover the properties required for such.
